# Predicting Survival of Metastatic Clear Cell Renal Cell Cancer Treated with VEGFR-TKI-Based Sequential Therapy

**DOI:** 10.3390/cancers16162786

**Published:** 2024-08-07

**Authors:** Javier C. Angulo, Gorka Larrinaga, David Lecumberri, Ane Miren Iturregui, Jon Danel Solano-Iturri, Charles H. Lawrie, María Armesto, Juan F. Dorado, Caroline E. Nunes-Xavier, Rafael Pulido, Claudia Manini, José I. López

**Affiliations:** 1 Clinical Department, Faculty of Medical Sciences, European University of Madrid, 28905 Getafe, Spain; 2 Biobizkaia Health Research Institute, 48903 Barakaldo, Spain; carolineelisabeth.nunes-xavier@bio-bizkaia.eus (C.E.N.-X.); rpulidomurillo@gmail.com (R.P.); joseignacio.lopez@biocrucesbizkaia.org (J.I.L.); 3 Department of Nursing, Faculty of Medicine and Nursing, University of the Basque Country (UPV/EHU), 48940 Leioa, Spain; 4 Department of Urology, Urduliz University Hospital, 48610 Urduliz, Spain; david.lecumberricastanos@osakidetza.eus (D.L.); anemiren.iturreguidelpozo@osakidetza.eus (A.M.I.); 5 Pathology Department, Cruces University Hospital, 48903 Barakaldo, Spain; jondanel.solanoiturri@osakidetza.eus; 6 Molecular Oncology Group, Biogipuzkoa Health Research Institute, 20014 San Sebastián, Spain; charles.lawrie@bio-gipuzkoa.eus (C.H.L.); maria.armestoalvarez@bio-gipuzkoa.eus (M.A.); 7 IKERBASQUE, Basque Foundation for Science, 48009 Bilbao, Spain; 8 Radcliffe Department of Medicine, University of Oxford, Oxford OX3 9DU, UK; 9 Sino-Swiss Institute of Advanced Technology (SSIAT), Shanghai University, Shanghai 201800, China; 10 PeRTICA Statistical Solutions, Plaza de la Constitución, 2, 28943 Fuenlabrada, Spain; jfdorado@pertica.es; 11 Department of Tumor Biology, Institute for Cancer Research, Oslo University Hospital Radiumhospitalet, 0379 Oslo, Norway; 12 Pathology Department, S. Giovanni Bosco Hospital, 10154 Turin, Italy; claudiamaninicm@gmail.com

**Keywords:** metastatic renal cell carcinoma, tyrosine kinase inhibitor sunitinib, nomogram, cancer-specific survival, prognosis, treatment response

## Abstract

**Simple Summary:**

Despite the continuous therapeutic efforts metastatic renal cell carcinoma (mRCC) is a dreadful disease, but the many options available provide an horizon of hope for these patients. Sequential therapy based on vascular endothelial growth factor-tyrosine kinase inhibitors (VEGFR-TKI) continues in use. We present a nomogram for a more individualized and accurate estimation of cancer-specific survival (CSS) for patients with clear-cell (CC) mRCC treated with nephrectomy and VEGFR-TK, based on four independent clinical predictors: Eastern Cooperative Oncology Group (ECOG) status; International Metastatic RCC Database Consortium (IMDC) score; Morphology, Attenuation, Size and Structure (MASS) and Response Evaluation Criteria in Solid Tumors (RECIST) response criteria. This tool may be useful to clinicians assessing risk and prognosis of patients with mRCC.

**Abstract:**

(1) Objective: To develop a clinically useful nomogram that may provide a more individualized and accurate estimation of cancer-specific survival (CSS) for patients with clear-cell (CC) metastatic renal cell carcinoma (mRCC) treated with nephrectomy and vascular endothelial growth factor receptor–tyrosine kinase inhibitor (VEGFR-TKI)-based sequential therapy. (2) Methods: A prospectively maintained database of 145 patients with mRCC treated between 2008 and 2018 was analyzed to predict the CSS of patients receiving sunitinib and second- and third-line therapies according to current standards of practice. A nomogram based on four independent clinical predictors (Eastern Cooperative Oncology Group status, International Metastatic RCC Database Consortium score, the Morphology, Attenuation, Size and Structure criteria and Response Evaluation Criteria in Solid Tumors response criteria) was calculated. The corresponding 1- to 10-year CSS probabilities were then determined from the nomogram. (3) Results: The median age was 60 years (95% CI 57.9–61.4). The disease was metastatic at diagnosis in 59 (40.7%), and 86 (59.3%) developed metastasis during follow-up. Patients were followed for a median 48 (IQR 72; 95% CI 56–75.7) months after first-line VEGFR-TKI initiation. The concordance probability estimator value for the nomogram is 0.778 ± 0.02 (mean ± SE). (4) Conclusions: A nomogram to predict CSS in patients with CC mRCC that incorporates patient status, clinical risk classification and response criteria to first-line VEGFR-TKI at 3 months is presented. This new tool may be useful to clinicians assessing the risk and prognosis of patients with mRCC.

## 1. Introduction

Despite renal cancer being a relatively infrequent neoplasia, its incidence has increased in the last few decades [1]. It is a urologic malignancy with poor prognosis, largely due to the incurability of metastatic disease [2], with the total number of cancer deaths increasing due to both aging and population growth [3]. However, as with leukemia and melanoma, metastatic renal cell carcinoma (mRCC) is one of the malignancies with outstanding therapeutic advances that may also account for mortality reduction [4].

The targeted therapy era of metastatic renal cancer started two decades ago, along with the relatively recent advent of immunotherapy and immune-oncology (IO) combination therapy in the field, vascular endothelial growth factor receptor–tyrosine kinase inhibitors (VEGFR-TKIs) have greatly contributed to the increased survival of this dreadful disease [5]. In the absence of specific molecular markers, many prognostic factors have been evaluated and identified for mRCC. Most are inherent to the patient (performance status) or tumor burden (cytoreductive nephrectomy, metastatic weight, biochemical and hematologic parameters) or treatment-related (therapeutic response, disease-free interval, time from first diagnosis to the development of metastatic disease and treatment tolerance and compliance) [6,7,8,9]. However, the continuous therapeutic advances in the field have made it difficult to universalize a prognostic model for all the different therapeutic scenarios. The risk stratification classification of the International Metastatic RCC Database Consortium (IMDC) has been the major criteria used for the selection of treatment during the last decade. Under this schema, antiangiogenic agents tend to be primarily used in patients with a more favorable prognosis, whereas patients with intermediate or poor risk tend to be treated with a combination of antiangiogenic agents and immunotherapy, targeting immune checkpoints inhibitors (ICIs) such as programmed cell death receptor (PD-1) or its ligand (PD-L1) or the cytotoxic T-lymphocyte antigen 4 (CTLA-4) receptor [10]. However, this treatment approach is not without controversy, and some of the most important trials upon which IO combinations were originally tested did not in fact show a significant benefit in the good prognosis group [11,12,13]. Consequently, VEGFR-TKI treatment continues to be used with a significant proportion of mRCC patients, and sequential treatment with targeted agents is highly recommended despite uncertainty surrounding what is the best sequence of agents for clinical use. The goal of treatment for mRCC is to prolong survival while maintaining a good quality of life, and in real-life clinical practice, this determines the choice of second-line and later-line agents [14]. Also, access to treatment options widely varies among health systems [15].

The objective of our study is to analyze the clinical and pathological variables that determine long-term survival in patients with clear-cell (CC) mRCC receiving sequential targeted therapy initiated by first-line VEGFR-TKI in a real-world setting. Based on these data, we developed a nomogram to predict survival that could also be useful to promote risk stratification and treatment planning and segregate patients at a higher risk of death that may need more aggressive treatment combinations with regards to their first-line therapy. Moreover, this analysis could be used as reference data to search for and validate new markers of prognosis in these patients.

## 2. Materials and Methods

### 2.1. Study Design and Patients

This is a non-interventional retrospective–prospective cohort multicenter study including patients with CC mRCC treated in two tertiary reference centers (Cruces and Donostia University Hospitals, Basque Country, Spain) treated with first-line VEGFR-TKI from 2008 to 2018. The study protocol was approved by the Institutional Ethics Committee (CEIm-Euskadi approval number PI2015059X, approval date 4 June 2015). All patients had radical nephrectomy, thus confirming the histopathological diagnosis of CC RCC. Metastases were diagnosed by imaging modalities, often confirmed by biopsy and sometimes surgical resection. The American Joint Committee on Cancer (AJCC) and National Comprehensive Cancer Network (NCCN) stages were used for tumor classification at the time of nephrectomy.

All patients received at least 1 cycle (4 weeks) of VEGFR-TKI (sunitinib, pazopanib or sorafenib) until progression or unresponsiveness. Response to therapy was assessed both with Response Evaluation Criteria in Solid Tumors (RECIST) and Morphology, Attenuation, Size, and Structure (MASS) criteria on the first contrast-enhanced computerized tomography (CECT) study after initiating therapy. Cases lost to follow-up were not eligible for the study, and patients with a diagnosis of non-clear-cell mRCC were also excluded. Participation in clinical trials was not considered a reason for exclusion. Patients were followed until death or the last follow-up (December 2022).

### 2.2. Assessments

Clinical characteristics of the patents were obtained from medical records and by a revision of the histopathology laboratory archive. There was confirmation of the histopathological data by two specialized pathologists (J.D.S-I. and J.I.L.). Performance status (PS) was evaluated using the Eastern Cooperative Oncology Group (ECOG) scale. Age, gender, stage at the initial diagnosis, the date of nephrectomy, the date of surgery of metastases, the number and site of metastases, the date of treatment initiation, the International Metastatic RCC Database Consortium (IMDC) risk criteria at the initiation of treatment, response according to RECIST and MASS criteria, the time and reason of discontinuation, the second- and third-line therapies used (also with response and length of each treatment), and the date of the last follow-up or date of death were evaluated. The cause of death was assessed individually for each patient by two independent observers (J.D.S-I. and D.L.). When disagreement existed regarding the cause of death, the decision was deferred to a third party (J.C.A.) according to the information provided in the clinical records. The effectiveness of treatment was analyzed based on the clinical and pathological criteria investigated. Safety was not evaluated in this study.

### 2.3. Statistical Analyses

Clinical and pathological characteristics were described using descriptive analytics. Differences between groups were compared with the chi-x2 test for qualitative measures and Student’s *t* test for quantitative measures. Overall survival (OS) and cancer-specific survival (CSS) were analyzed with the log-rank test. Multivariate Cox regression analysis for CSS was performed, including the prognostic factors significant in univariate analysis, to adjust for covariates. The significance value cut-off was *p* < 0.05 for the results. A nomogram to predict CSS using the independent variables identified is proposed and internally validated by bootstrapping. The accuracy of the predictive model is provided [16]. Statistical analysis was performed using Statistical Analysis System 9.4 (SASS Institute Inc., Cary, NY, USA) and the R Project for Statistical Computing (free software environment for statistical computing and graphics; version 3.5.0; http://www.r-project.org).

## 3. Results

### 3.1. Patients Characteristics at the Time of Nephrectomy

A total of 170 patients with CC mRCC treated with sunitinib as the first-line treatment were considered for the study. However, 25 were ineligible either because the response criteria could not be determined (*n* = 5), histology was not consistent with the diagnosis of CC (*n* = 4) or they were lost to follow-up (*n* = 16). Therefore, 145 patients were finally included into the study and followed until death or December 2022.

In this cohort, the male-to-female ratio was 2.5:1, with 104 (71.7%) males and 41 (28.3%) females. The median age of the patients at the time of the diagnosis of metastatic disease was 59 (IQR 51.5–66.5, range 25–82) years. Nephrectomy was performed in all patients. The median tumor size was 8 (IQR 5.7–10.3, range 3–20) cm. The Fuhrman grade was 1 in 6 (4.1%), 2 in 29 (20%), 3 in 44 (30.4%), and 4 in 66 (45.5%). For the AJCC T category, 22 patients (15.2%) were pT1, 18 (12.4%) pT2, 96 (66.2%) pT3, and 9 (6.2%) pT4. At the time of nephrectomy, the NCCN stage was I in 18 (12.4%), II in 10 (6.9%), III in 51 (35.2%), and IV in 66 (45.5%). Positive nodes were identified in 23 (15.9%) of cases, with a single positive node (N1) in 15 (65.2%) and several (N2) in 8 (34.8%). Metastatic disease was present at the time of diagnosis in 59 (40.7%) patients, 15 (25%) with single and 44 (75%) with multiple metastasis. In 86 (59.3%), metachronous metastasis developed at a median of 20 (IQR 42, range 1–201) months after nephrectomy. Metastases were pathologically confirmed in 32 cases (22%) and surgical resected in 12 (8.3%).

### 3.2. Patients Characteristics at Initiation of Treatment of mRCC

The median age of the patient at the time of treatment was 60 (IQR 14, 95% CI 57.9–61.4) years. The ECOG scale and IMDC risk classification are depicted in Table 1. Treatment was initiated in all cases after a diagnosis of mRCC. Patients were followed for a median of 48 (IQR 72; 95% CI 56–75.7) months after sunitinib initiation.

### 3.3. Response to First-Line Treatment

Table 2 presents the classification of response to VEGFR-TKI at 3 months. At a median 17 ± 26.4 months, disease progression was confirmed in 129 patients (89%). At the last follow-up, eight cases (5.5%) continued the initial treatment. Reasons for discontinuation were ineffectiveness for 86 (59.3%), intolerance for 33 (22.8%), death for 12 (8.3%) and other reasons for 6 (4.1%).

### 3.4. Other Treatments Received

Second-line therapy was used in 89 patients (59.3%) and consisted of small-molecule TKI axitinib or multi-TKI cabozantinib (*n* = 56), a mammalian target of rapamycin (mTOR) inhibitors everolimus or temsirolimus (*n* = 23), and ICIs nivolumab and/or ipilimumab (*n* = 10). Third-line therapies were used in 30 patients (20%) and consisted of multi-TKI cabozantinib (*n* = 11), mTOR inhibitor temsirolimus (*n* = 7), and a combination of ICIs plus TKI (pembrolizumab or avelumab plus axitinib) (*n* = 12). Rechallenge with TKI as the fourth-line treatment was used in nine patients (6.2%), all with a duration of tumor control ≥ 6 months on the first-line therapy.

### 3.5. Overall and Cancer-Specific Survival

**Figure 1:**
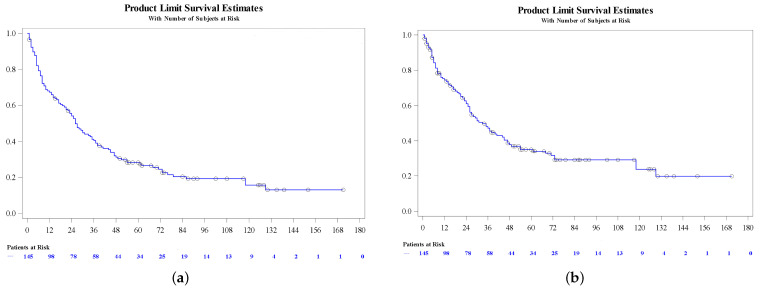
(**a**) Overall survival and (**b**) cancer-specific survival with VEGFR-TKI sequential therapy.

Survival (OS and CSS) at different times is presented in Table 3 and in Figure 1.

**Figure 2:**
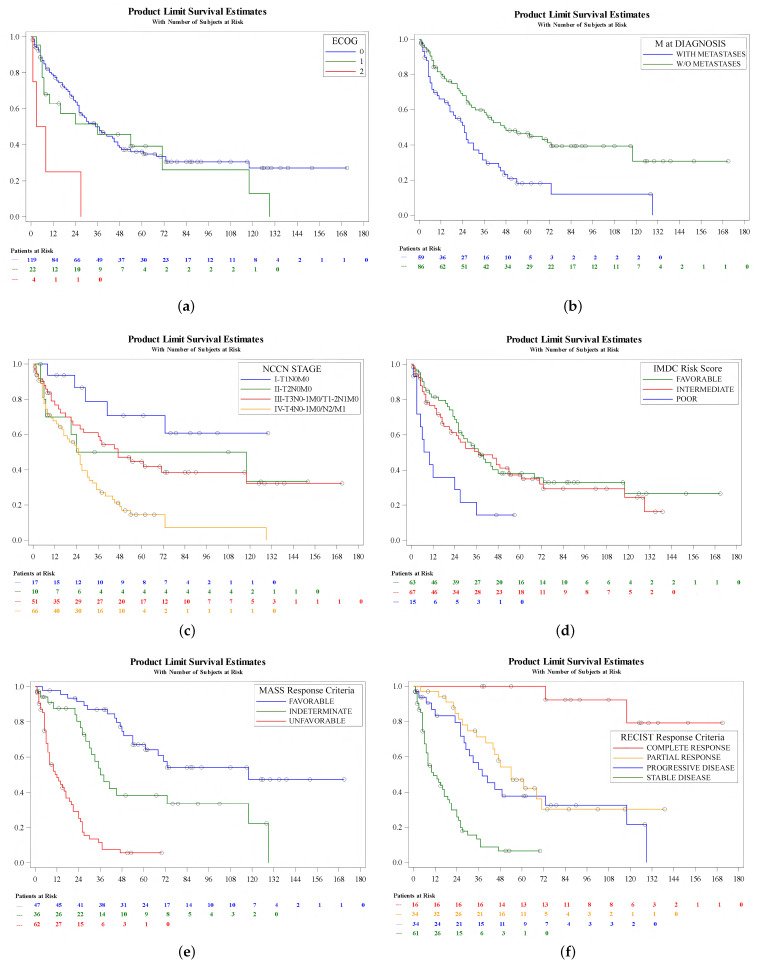
CSS according to variables predictive in univariate analysis: (**a**) ECOG status; (**b**) metastases at diagnosis; (**c**) NCCN stage at diagnosis; (**d**) IMDC risk score at initiation of treatment; (**e**) MASS response criteria; (**f**) RECIST response criteria.

A Kaplan–Meier analysis of CSS was performed. Variables predicting prognosis included ECOG status (log-rank, *p* = 0.004), metastasis at diagnosis (log-rank, *p* = 0.0004), NCCN stage at diagnosis (log-rank, *p* = 0.0001), IMDC risk classification at initiating treatment (log-rank, *p* = 0.005), MASS response criteria (log-rank, *p* < 0.0001) and RECIST response criteria (log-rank, *p* < 0.0001) (Figure 2). Patient age (log-rank, *p* = 0.1), gender (log-rank, *p* = 0.2), pT category (log-rank, *p* = 0.1), pN category (log-rank, *p* = 0.09) and Fuhrman grade (log-rank, *p* = 0.5) at the time of diagnosis were not determinants of prognosis in this series.

Table 4 shows the corresponding hazard ratios and confidence interval limits for each variable by univariate analysis. Patient age, ECOG performance status, the synchronicity of metastases, the NCC stage, the IMDC risk group, and the MASS and RECIST response criteria at 3 months were significant (*p* < 0.05). Multivariate analysis revealed that ECOG performance status (0–1 vs. 2, HR 3.36 (95% C.I. 1.88–5.97); *p* = 0.0004), the RECIST of first-line therapy at 3 months (stable and partial response vs. complete response, HR 7.1 (95% C.I. 1.58–31.99); progression vs. complete response, HR7.46 (95% C.I. 1.07–52.07); *p* = 0.008), MASS response criteria at 3 months (1 vs. 3, HR 0.16 (95% C.I. 0.04–0.61); 2 vs. 3, HR 0.25 (95% C.I. 0.07–0.9); *p* < 0.0001), and the IMDC risk group (poor vs. favorable and intermediate, HR 2.09 (95% C.I. 1.07–4.07); *p* = 0.028) stayed as independent factors (*p* < 0.05) of CSS after VEGFR-TKI-based sequential therapy.

### 3.6. Nomogram for the Prediction of Prognosis

**Figure 3:**
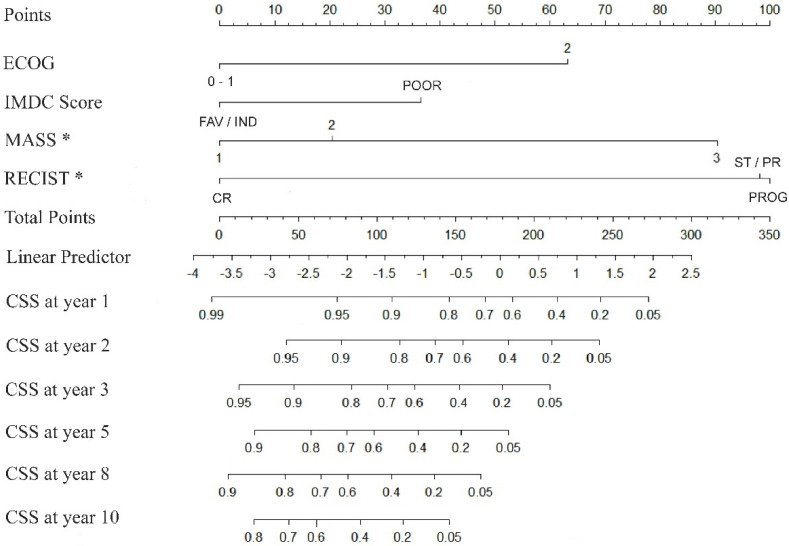
Nomogram predicting the probability of CSS at different times (1 to 10 years), calculated by obtaining the value for each parameter by drawing a straight line to the point axis, adding the points together, and filling the sum-of-total-points axis (* Response Criteria).

**Figure 4:**
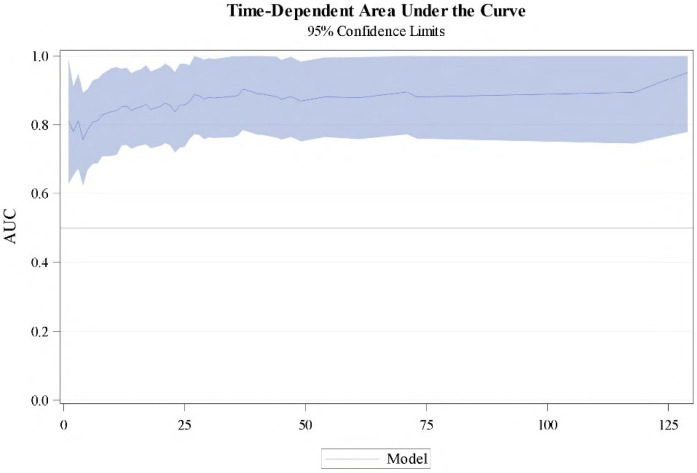
Area under the curve for the Cox predictive model with 95% CI during follow-up.

Using the multivariate Cox model presented, a nomogram-predicted 1 to 10 years CSS probability was generated (Figure 3). The model was internally validated using 500 bootstrap samples, and the concordance was 0.778 (95% CI 73.3–81.6%). The time-dependent area under the curve (AUC) based on 50 perturbed samples is represented for CSS at different times of the follow-up (Figure 4).

## 4. Discussion

The survival of mRCC patients has vastly improved since the advent of targeted therapy [14]. In real-life settings, VEGFR-targeted agents such as sunitinib, sorafenib, and pazopanib have been widely used in the last two decades. Axitinib and cabozantinib, often considered after the failure of sunitinib, have also consolidated the sequential use of VEGFR-TKI-based treatment. Everolimus and, more recently, nivolumab have been used after disease progression with first-line sunitinib or pazopanib as well [15]. The prolonged survival of patients with mRCC who received sequential targeted agents has been demonstrated in many clinical trials, but the optimal sequences remain unidentified. However, clinical trials are not always representative of the real-life population [17].

The landscape for the sequential treatment of mRCC has become more diverse with the advent of immunotherapy, thus complicating the definition of the optimal treatment succession. However, several lessons have been learnt. On one hand, the benefit of the ICI nivolumab after VEGFR-TKIs did not depend on the prior therapy, the number of antiangiogenic drugs used, or the duration of response [14]. On the other, the discovery and approval of ICIs has revolutionized the management of mRCC, and several ICI-based combinations have become the new standard of care for these patients [10]. Options have expanded in recent years to include as the standard first-line therapy the combinations of the IO agents ipilimumab and nivolumab and VEGFR-targeted therapy with IO agents [18]. Nonetheless, monotherapy with antiangiogenic TKIs (e.g., pazopanib or sunitinib) still represents a first-line treatment option for selected patients in the favorable-risk group according to the IMDC model, and ICI monotherapy with the anti-PD-1 nivolumab is currently the main second-line option. For third-line and subsequent therapies, this typically involves rechallenging a drug that previously achieved tumor control for 6 month or longer, and in cases in which treatment was stopped due to toxicity, this is another alternative [17].

Different combination therapies tend to be used for intermediate- and poor-risk groups. Both the heterogeneity of RCC and its constantly changing therapeutic scenario have complicated the establishment of prognostic markers [19,20]. Other continued controversies have also contributed to this issue, such as whether cytoreductive nephrectomy is valuable and/or how to optimize managements of adverse effects to minimize dose reduction and treatment discontinuation [21,22,23]. To make matters worse, not all patients have access to novel immunotherapy-based combinations [24].

We present a nomogram to predict long-term survival in patients with mRCC treated with first-line VGFR-TKI sunitinib, sorafenib or pazopanib and with successive treatment options including cabozantinib, everolimus or nivolumab. This graphic prediction tool takes into account factors that have an independent impact on outcome. These are the ECOG status and the IMDC risk score at the time of treatment initiation and both RECIST and MASS response criteria upon the first CECT study performed after initiating therapy.

The IMDC prognostic model was also developed and validated in patients with mRCC receiving VEGF-TKIs based on six prognostic criteria: time from the initial diagnosis to systemic therapy < 1 year, Karnofsky performance status < 80, serum hemoglobin, platelet count, absolute neutrophil count, and corrected serum calcium [9,25]. Patients are characterized as having favorable (no criteria), intermediate (1–2 criteria), or poor (≥3 criteria) risk. It is widely admitted that the IMDC model provides essential information to guide treatment decisions and also predict the effectiveness of systemic therapy and prognosis [15,26]. There is an interesting discussion about whether an assessment of the therapeutic response in mRCC on CECT for changes in morphology, attenuation, size, and structure according to the MASS criteria is more accurate than a response assessment based on the RECIST criteria widely adopted by academic institutions, cooperative groups and in clinical trials [27,28]. Our study supports that both criteria are valid and complementary to assess treatment response and also that a response to first-line VGFR-TKI has a clinical impact on CSS in the long term [29].

Several nomograms have been proposed to predict the prognosis of RCC but do not specifically refer to metastatic disease [30,31,32,33]. Others refer to advanced disease specifically treated with sunitinib [34] and pazopanib [35]. They use biochemical and hematologic parameters together with other interesting parameters like the topography of metastases and time from diagnosis to therapy. More recently, some nomograms have been developed to specifically evaluate prognosis in mRCC [36,37,38]. They differ from our model in that they do not include patients treated uniformly with targeted therapies and also in that nephrectomy or cancer-directed surgery is not performed in all cases either. Interestingly, these nomograms uniformly use the topographic distribution of metastases as prognostic factors involved and, unlike the one we propose, the evaluation of response to first-line VEGFR-TKI was not included.

In light of the literature findings, the main limitation of our model is that the topographic distribution of metastases is not evaluated. Another limitation of this model stands on the fact that it is based on data from two institutions, and external validation is advisable before it can be generalized. Additionally, variables such as the topography of metastases and VEGFR-TKI dose reduction or discontinuation due to adverse effects are not considered and could have prognostic value [23,39]. Similarly, the number of patients who received fourth-line or further therapies is not considered. On the other hand, the main strength of the model is that it is based on simple measurements of clinical importance, such as patient status baseline (ECOG scale), the risk category of metastatic disease before treatment (IMDC classification) and a concise assessment of response after initiating therapy (the RECIST and MASS criteria). These variables assessed the baseline and first months of treatment initiation to determine patient prognosis in the long term. A durable complete response can be observed regardless of the prognostic group [40]. Nephrectomy was always performed in these patients. A limitation may also exist if this nomogram is used in a population of mRCC patients that do not receive nephrectomy. We cannot either assume the value of this model in patients receiving sequential treatment initiated with upfront immunotherapy [41,42]. This study serves to evaluate the long-term survival of mRCC in the clinical practice and seems a good starting point to investigate immunohistochemical and molecular makers predictive of prognosis in this series of patients.

## 5. Conclusions

VEGFR-TKI sequential therapy has greatly contributed to ameliorate the survival of this uncurable disease in the past few years and is still used today to some extent, especially in patients with a presumed favorable prognosis. Hopefully, the identification of prognostic biomarkers will allow a better selection of individualized therapy, given the many options available today. In the meantime, the nomogram proposed here may be a useful prognostic tool, at least for clinicians with patients initiating sunitinib or pazopanib as first-line therapy. The baseline condition, IMDC risk classification and the evaluation of therapeutic response upon the first CECT study after initiating therapy, using both RECIST and MASS criteria, is a simple and reliable way to predict long-term prognosis.

Our experience favors the observation that sequential treatment with targeted agents improves the survival of CC mRCC and also that treatment should be continued until disease progression or even beyond in the targeted therapy era. However, it remains to be studied whether this nomogram could be used to promote immunotherapy or IO combination therapy after an initial course of VEGFR-TKI therapy.

## Figures and Tables

**Table 1:** Clinical and pathological variables of mRCC patients.

	Clinical Data	Total Series (*n* = 145)
ECOG Scale ^1^	0, *n* (%)	119 (82)
	1, *n* (%)	22 (15.2)
	2, *n* (%)	4 (2.8)
IMDC ^2^	Favorable, *n* (%)	63 (43.4)
	Intermediate, *n* (%)	67 (46.2)
	Poor, *n* (%)	15 (10.4)

^1^ Eastern Cooperative Oncology Group; ^2^ International Metastatic RCC Database Consortium.

**Table 2:** Response to VEGFR TKI sunitinib at 3 months according to different criteria.

	First-Line Treatment Response	Total Series (*n* = 145)
RECIST ^1^	Complete Response, *n* (%)	16 (11)
	Partial Response, *n* (%)	34 (23.5)
	Stable Disease, *n* (%)	34 (23.5)
	Progression, *n* (%)	61 (42)
MASS ^2^	Favorable Response, *n* (%)	47 (32.4)
	Indeterminate Response, *n* (%)	36 (24.8)
	Unfavorable Response, *n* (%)	62 (42.8)

^1^ Response Evaluation Criteria in Solid Tumors; ^2^ Morphology, Attenuation, Size, and Structure.

**Table 3:** Overall survival (OS) and cancer-specific survival (CSS) at different follow-up times ^1^.

Time	OS (% Surviving, 95% CI)	CSS (% Surviving, 95% CI)
1 year	67.4 (59.1–74.4)	74.5 (66.3–81)
2 years	54 (45.5–61.8)	61.1 (52.2–68.8)
3 years	40.5 (32.4–48.5)	47.2 (38.4–55.6)
5 years	28.2 (21–35.8)	35.1 (26.743.5)
8 years	19.3 (12.8–26-8)	29 (20.8–37.7)
10 years	15.8 (9.4–23.7)	23.7 (14.8–33.8)

^1^ Calculated after initiation of first-line VEGFR-TKI sunitinib.

**Table 4:** Cox regression model to predict cancer-specific survival of patients with mRCC.

Univariate Analysis	Hazard Ratio	95% CI	*p*-Value
Female vs. male	1.33	0.85–2.09	0.21
Age ≤60 years vs. >60 years	0.723	0.47–1.09	0.13
ECOG ^1^ 0 vs. 1	0.8	0.46–1.4	**0.01**
ECOG 0 vs. 2	0.21	0.08–0.59	
ECOG 1 vs. 2	0.27	0.09–0.82	
pT1 vs. pT2	0.55	0.22–1.37	0.13
pT1 vs. pT3	0.47	0.23–0.98	
pT1 vs. pT4	0.31	0.11–0.86	
pT2 vs. pT3	0.86	0.45–1.63	
pT2 vs. pT4	0.56	0.22–1.47	
pT3 vs. pT4	0.66	0.3–1.44	
pN0 vs. pN1	1.06	0.48–2.3	0.11
pN0 vs. pN2	0.44	0.2–0.95	
pN1 vs. pN2	0.41	0.14–1.18	
Synchronous vs. metachronous metastases	2.07	1.36–3.15	**0.0006**
Stage I vs. II	0.45	0.14–1.47	**0.0004**
Stage I vs. III	0.45	0.17–1.16	
Stage I vs. IV	0.21	0.08–0.52	
Stage II vs. III	1.0	0.41–2.43	
Stage II vs. IV	0.46	0.2–1.1	
Stage III vs. IV	0.46	0.29–0.75	
Fuhrman grade 1 vs. 2	1.32	0.44–3.97	0.55
Fuhrman grade 1 vs. 3	1.07	0.37–3.08	
Fuhrman grade 1 vs. 4	0.87	0.31–2.42	
Fuhrman grade 2 vs. 3	0.82	0.43–1.53	
Fuhrman grade 2 vs. 4	0.66	0.36–1.19	
Fuhrman grade 3 vs. 4	0.81	0.49–1.31	
IMDC ^2^ risk poor vs. favorable	2.77	1.43–5.35	**0.009**
IMDC risk poor vs. intermediate	2.45	1.28–4.69	
IMDC risk favorable vs. intermediate	0.88	0.57–1.38	
MASS ^3^ criteria 1 vs. 2	0.43	0.23–0.8	**<0.0001**
MASS criteria 1 vs. 3	0.11	0.06–0.19	
MASS criteria 2 vs. 3	0.25	0.14–0.43	
RECIST ^4^ stable vs. progression	0.25	0.14–0.45	**<0.0001**
RECIST stable vs. complete response	11.53	2.66–49.91	
RECIST stable vs. partial response	1.27	0.67–2.4	
RECIST progression vs. complete response	45.16	10.44–195.43	
RECIST progression vs. partial response	4.99	2.85–8.75	
RECIST complete vs. partial response	0.11	0.02–0.48	
**Multivariate Analysis**	**Hazard Ratio**	**95% CI**	***p*-value**
ECOG 0–1 vs. 2	3.36	1.88–5.97	**0.0004**
IMDC risk poor vs. favorable–intermediate	2.09	1.07–4.07	**0.028**
MASS criteria 1 vs. 3	0.16	0.04–0.61	**<0.0001**
MASS criteria 2 vs. 3	0.25	0.07–0.9	
RECIST stable–partial response vs. complete response	7.1	1.58–31.99	**0.008**
RECIST progression vs. complete response	7.46	1.07–52.07	

^1^ Eastern Cooperative Oncology Group; ^2^ International Metastatic RCC Database Consortium; ^3^ Morphology, Attenuation, Size, and Structure; ^4^ Response Evaluation Criteria in Solid Tumors. Values in bold are statistically significant.

## Data Availability

Full data will be provided by the corresponding author upon reasonable request.

## Abbreviations

AJCC: American Joint Committee on Cancer; AUC: area under the curve; CC: clear cell; CECT: contrast-enhanced computerized tomography; CI: confidence interval; CSS: cancer-specific survival; CTLA-4: cytotoxic T-lymphocyte antigen 4; ECOG: Eastern Cooperative Oncology Group; HR: hazard ratio; ICI: immune checkpoints inhibitor; IMDC: International Metastatic RCC Database Consortium; IQR: interquartile range; IO: immune-oncology; MASS: Morphology, Attenuation, Size, and Structure; mRCC: metastatic renal cell carcinoma; NCCN: National Comprehensive Cancer Network; OS: overall survival; PD-1: programmed cell death receptor; PD-L1: programmed cell death receptor ligand; RECIST: Response Evaluation Criteria in Solid Tumors; OS: overall survival; PS: performance status; SE: standard error; VEGFR-TKI: vascular endothelial growth factor receptor–tyrosine kinase inhibitor.
